# Supplementary material for: Risk Assessment and Determination of Heavy Metals in Home Meal Replacement Products by Using Inductively Coupled Plasma Mass Spectrometry and Direct Mercury Analyzer
Source: Foods. 2022 Feb 10;11(4):504. doi: 10.3390/foods11040504 (PMC8870816; doi:10.3390/foods11040504)
Supplement: Supplementary file 1 [file foods-11-00504-s001.zip › Table S1 (revised).pdf]

**Table S1. Recovery of the six heavy metals.**

| Matrix type | Heavy metal element | Recovery (%)[a] | | |
|---|---|---|---|---|
| Non-fatty solid phase | Pb (mg/kg) | 98.96±0.53 | 97.29±1.03 | 95.21±2.14 |
| | Cd (mg/kg) | 89.06±2.20 | 91.02±2.04 | 90.96±5.37 |
| | As (mg/kg) | 99.01±5.46 | 101.82±1.11 | 103.56±8.50 |
| | Sn (mg/kg) | 104.23±0.53 | 109.78±8.43 | 111.97±14.78 |
| | Hg (μg/kg) | 115.65±4.04 | 118.02±3.31 | 112.02±5.82 |
| | Me-Hg (μg/kg) | 80.97±3.12 | 85.06±4.62 | 94.56±4.25 |
| Fatty solid phase | Pb (mg/kg) | 97.86±4.20 | 90.34±1.05 | 80.65±0.65 |
| | Cd (mg/kg) | 83.23±2.10 | 85.67±9.45 | 90.78±6.65 |
| | As (mg/kg) | 99.02±0.54 | 101.23±1.04 | 104.65±2.15 |
| | Sn (mg/kg) | 107.98±5.38 | 111.92±2.03 | 114.86±2.19 |
| | Hg (μg/kg) | 116.78±8.49 | 112.65±1.10 | 104.65±5.45 |
| | Me-Hg (μg/kg) | 102.23±14.68 | 113.23±8.42 | 119.87±0.52 |
| Non-fatty liquid phase | Pb (mg/kg) | 83.06±5.83 | 85.87±3.30 | 89.06±4.03 |
| | Cd (mg/kg) | 84.02±4.21 | 90.34±4.61 | 94.54±3.11 |
| | As (mg/kg) | 100.29±2.06 | 105.76±5.76 | 108.87±9.04 |
| | Sn (mg/kg) | 110.64±1.23 | 115.23±1.46 | 116.76±2.97 |
| | Hg (μg/kg) | 112.54±7.05 | 99.07±8.52 | 93.65±6.13 |
| | Me-Hg (μg/kg) | 93.02±3.07 | 99.78±1.20 | 103.45±2.85 |
| Fatty liquid phase | Pb (mg/kg) | 94.02±4.56 | 108.96±5.02 | 103.23±0.76 |
| | Cd (mg/kg) | 100.29±10.26 | 98.76±13.91 | 87.56±4.56 |
| | As (mg/kg) | 101.87±7.84 | 104.72±8.21 | 112.96±5.23 |
| | Sn (mg/kg) | 114.79±2.67 | 118.02±5.01 | 105.02±0.87 |
| | Hg (μg/kg) | 95.47±7.41 | 89.76±4.03 | 90.78±1.78 |
| | Me-Hg (μg/kg) | 92.87±3.51 | 84.01±5.40 | 88.93±4.30 |

[a]Recovery was evaluated with 0.25, 1.0, 10.0 mg/kg for Pb, Cd, As, and Sn and 5, 20, 100 μg/kg for Hg and Me-Hg spiked concentrations and are shown as mean ± relative standard deviation (*n*=3).
