# Supplementary material for: Risk Assessment and Determination of Heavy Metals in Home Meal Replacement Products by Using Inductively Coupled Plasma Mass Spectrometry and Direct Mercury Analyzer
Source: Foods. 2022 Feb 10;11(4):504. doi: 10.3390/foods11040504 (PMC8870816; doi:10.3390/foods11040504)
Supplement: Supplementary file 1 [file foods-11-00504-s001.zip › Table S2 (revised).pdf]

Table S2. The μ(Rec), RSU, CSU, and EU of the heavy metals.

Heavy metal element	μ(Rec)	RSU	CSU	EU
Pb (mg/kg)	0.167	0.168	0.17	$\mu(C_{Pb}) = C_{Pb} \times 0.34$
Cd (mg/kg)	0.093	0.093	0.10	$\mu(C_{Cd}) = C_{Cd} \times 0.20$
As (mg/kg)	0.155	0.153	0.16	$\mu(C_{As}) = C_{As} \times 0.32$
Sn (mg/kg)	0.321	0.320	0.33	$\mu(C_{Sn}) = C_{Sn} \times 0.66$
Hg (μg/kg)	0.082	0.084	0.08	$\mu(C_{Hg}) = C_{Hg} \times 0.16$
Me-Hg (μg/kg)	0.198	0.204	0.20	$\mu(C_{Me-Hg}) = C_{Me-Hg} \times 0.40$
