# Supplementary material for: Risk Assessment and Determination of Heavy Metals in Home Meal Replacement Products by Using Inductively Coupled Plasma Mass Spectrometry and Direct Mercury Analyzer
Source: Foods. 2022 Feb 10;11(4):504. doi: 10.3390/foods11040504 (PMC8870816; doi:10.3390/foods11040504)
Supplement: Supplementary file 1 [file foods-11-00504-s001.zip › Table S3 (revised).pdf]

Table S3. Total average food intake data and 95th percentile food intake data for the six heavy metals in HMR (g/day).

Matrix type	Six heavy metals	
	Total average food intake data (g/day)	95th percentile food intake data (g/day)
Non-fatty solid phase	0.041	0.134
Fatty solid phase	0.018	0.035
Non-fatty liquid phase	0.030	0.010
Fatty liquid phase	0.001	0.009
