# Supplementary material for: Risk Assessment and Determination of Heavy Metals in Home Meal Replacement Products by Using Inductively Coupled Plasma Mass Spectrometry and Direct Mercury Analyzer
Source: Foods. 2022 Feb 10;11(4):504. doi: 10.3390/foods11040504 (PMC8870816; doi:10.3390/foods11040504)
Supplement: Supplementary file 1 [file foods-11-00504-s001.zip › Table S4.pdf]

Table S4. Average body weights and exposure periods for calculating chronic daily intake in HMR.

Ages	Average body weight (kg)	Body weight (kg)	Exposure period (year)
Above 65	58.5	57.9	16.5
20-64		64.4	45
13-19		59.1	7
7-12		37.4	6
3-6		19.5	4
1-2		12.5	2
