# Supplementary material for: Risk Assessment and Determination of Heavy Metals in Home Meal Replacement Products by Using Inductively Coupled Plasma Mass Spectrometry and Direct Mercury Analyzer
Source: Foods. 2022 Feb 10;11(4):504. doi: 10.3390/foods11040504 (PMC8870816; doi:10.3390/foods11040504)
Supplement: Supplementary file 1 [file foods-11-00504-s001.zip › Table S5.pdf]

Table S5. Risk and margin of exposure of six heavy metals in HMR.

Matrix type	Risk and margin of exposure	
	Total average risk and margin of exposure	95th percentile risk and margin of exposure
Non-fatty solid phase	9.67×10^{6}	2.94×10^{6}
Fatty solid phase	2.68×10^{7}	1.39×10^{7}
Non-fatty liquid phase	2.17×10^{7}	6.22×10^{7}
Fatty liquid phase	5.34×10^{8}	5.94×10^{7}
